# Non-diabetic Kidney Disease in Diabetic Population: A Single-Center Study From South India

**DOI:** 10.7759/cureus.23899

**Published:** 2022-04-06

**Authors:** Mahesh Eswarappa, Saritha Suryadevara, Rajashekar R, Mahesh Kumar K B, Gurudev K C, Puru Tyagi, Avin V

**Affiliations:** 1 Nephrology, Ramaiah Medical College, Bangalore, IND; 2 Nephrology, Amsa Renal Care, Mount Fleuri, SYC; 3 Medicine, Jagadguru Jayadeva Murugarajendra (JJM) Medical College, Davanagere, IND

**Keywords:** dm, diabetes mellitus, dkd, diabetic kidney disease, nkdk, non-diabetic kidney disease, renal failure, renal biopsy

## Abstract

Introduction: Diabetic kidney disease (DKD) is the commonest cause of chronic kidney disease and end-stage kidney disease worldwide, consequently it has become an important productive implication to the healthcare system. This study was conducted to assess the prevalence of non-DKD (NDKD) in diabetic patients from south India.

Objective: To assess the prevalence of NDKD in type 2 diabetes mellitus patients presenting to a tertiary care hospital from south India and also to analyze clinical clues to establish a diagnosis of NDKD.

Patient and methods: It is a retrospective observational study of analyzing patient characteristics and renal biopsies. All Diabetic patients with a clinical suspicion of non-diabetic kidney disease who underwent renal biopsy during the study period between January 2012 and June 2017 were included. Based on the biopsy findings, the patients were classified into three groups (isolated diabetic nephropathy, isolated NDKD, and NDKD with underlying diabetic nephropathy) and patients’ characteristics were compared between the groups for analysis.

Results: A total of 236 renal biopsies were analyzed for the study. Of that, 114 had features of DKD, 78 NDKD with diabetic nephropathy (DN) and 44 had isolated NDKD. Acute interstitial nephritis was the most common cause of NDKD.

Conclusion: From the current study, the long duration of diabetes mellitus beyond five years and hypertension beyond two years reasonably predict DKD.

## Introduction

Diabetic kidney disease (DKD) is defined as a clinical syndrome in a patient with diabetes mellitus (DM) characterized by persistent albuminuria (>300 mg/day or >200 μg/min) on at least two occasions 3-6 months apart, elevated blood pressure and progressive decline in renal function. DKD is the commonest cause of chronic kidney disease and end-stage kidney disease worldwide [1]; consequently, it has become an important productive implication to the healthcare system. Around 20%-40% of patients with DM develop DKD [2]. However, there are a substantial number of diabetic patients who are diagnosed with non-diabetic kidney disease (NDKD), reaching as high as 64%-85% in some studies of biopsied patients [3-11]. The diagnosis of DKD in a bulk of the patients remains clinical and invasive tests like renal biopsy are usually reserved for atypical cases. While such an approach is well validated in type 1 DM, a rethinking of this approach may be required in type 2 DM given the often high prevalence of NDKD [4,5].

NDKD warrants special attention because when picked up early, interventions can often reduce the associated morbidity, mortality, or quality of life. The presence of biopsy-demonstrated NDKD in patients with diabetes varies from 8% to 85% in the published literature [5-8]. The etiological and demographic data is sparse from South India. Hence this study was conducted to evaluate the percentage of NDKD in diabetic patients from south India.

## Materials and methods

It is a retrospective observational study analyzing patient characteristics and renal biopsies done on diabetic patients who had an atypical presentation. Indicators of kidney biopsy in diabetic patients are shown in Table 1. All diabetic patients with a clinical doubt of NDKD who underwent renal biopsy during the study period between January 2012 and June 2017 were included in the study. Institutional ethics committee (Ramaiah Medical College; ECR/215/Inst/KA/2013/RR-19) approval was obtained prior to the study. Patient features consist of demographic parameters, years of diabetes, duration of hypertension (years), blood pressure (mm hg), occurrence of diabetic retinopathy, HbA1c (%), hematuria, 24-hour urinary protein (mg/day), creatinine (mg/dL), blood urea nitrogen (BUN, mg/dL), albumin (g/L), triglyceride (mg/dL), total cholesterol (mg/dL), blood pressure, and complement level (wherever available) along with the renal biopsy results were derived from hospital records for analysis. Depending on the histological finding, the cases were divided into three groups: group I (DKD only: N=114), group II (NDKD with underlying DKD; N=78), and group III (isolated NDKD; N=44). Patient characteristics were compared between the groups for analysis.

**Table 1:** Indications for renal biopsy

Indications
Unexplained renal failure
Overt proteinuria without retinopathy
Active urine sediment
Rapidly progressive decline in GFR
Presence of systemic features
Short duration of diabetes

The data were analyzed using WPS software and JMP Statistics. Numerical data were interpreted as mean ± standard deviation. Positive or negative values were interpreted as count/total sample number. Correlation between groups was analyzed using the Chi-square test, analysis of variance, unpaired t-test, and nonparametric test. Binary univariate and multivariate regression analyses were utilized to analyze the relationship between baseline characteristics and outcomes. A P-value < 0.05 was considered statistically significant.

## Results

A total of 236 diabetic pts in whom renal biopsies were done for atypical features were analyzed for the study. Among these, 114 biopsies showed features of DKD, 78 had features of NDKD overlapped with DKD and 44 biopsies had features of isolated NDKD. We found a definitive yield based on clinical suspicion of 51.69% - 122 out of 236 in our study. The usual indicators for renal biopsy in our study were an unexplained renal failure - 33.4% not corroborating with the clinical scenario, followed by nephrotic range proteinuria being 28.35%, proteinuria without retinopathy of 18.14%, and active urine sediment of 16.08% ( Table 1). Our study incorporated active urinary sediments as an indication for biopsy. A meta-analysis in 2013 also emphasized the importance of dysmorphic RBCs in predicting NDKD in diabetic patients [9].

The patient characteristics were analyzed between the groups to identify the clinical clues that could possibly suggest Non-Diabetic Kidney Disease. Table 2 details these characteristics. Age, sex, mean blood pressure, and glycemic control were comparable between the groups. 72% of the patients were hypertensive while the rest were nonhypertensive. The years of diabetes and hypertension were notably higher in the class with DKD, with a median of six years and two years, respectively. Unexplained renal failure was more frequent in the non-diabetic group with an incidence of 56.8%. Interestingly the incidences of azotemia, hypoalbuminemia, hypertriglyceridemia, anemia, and active urinary sediments were not significantly different between the groups. It may account for the observation that microscopic hematuria is seen in up to 30% of patients with diabetic kidney disease [10]. Surprisingly, the occurrence of diabetic retinopathy was near between the groups, and mean blood pressure was not meaningfully distinct between diabetic and non-diabetic groups (Table 2).

**Table 2:** Patient characteristics between the groups

Patient Characterstics	Diabetic Nephropathy (N= 114)	Diabetic Nephropathy With Non Diabetic Kidney Disease (N=78)	Non Diabetic Kidney Disease (N= 44)	P Value
Mean Age in Years	54.18	54.32	52.32	0.614
Median Duration Of Diabetes In Years	6	4	4	0.002
Median Duration Of Hypertension in Years	2	1	1	0.036
Unexplained Renal Failure	21.2%	37.5%	56.8%	<0.0001
Mean Blood Pressure (mmHg)	143	139	140	0.557
Median Serum Creatinine-(mg/dl)	3	3	2	0.820
Median Blood Urea mg/dl)	37.5	35	34	0.059
Haematuria	10.25%	14.76%	18.29%	0.090
Mean Haemoglobin(g/dl)	10.6	10.6	10.39	0.866
Mean Serum Albumin(g/dl)	3.5	3	3	0.455
Mean Total Cholestrol (mg/dl)	174	134	116	0.559
Mean Triglyceride (mg/dl)	150	158	248	0.457
Median Hba1c%	8.5	8.5	8	0.739
No Diabetic Retinopathy	82.5%	79%	84.1%	0.741
Dialysis, Transplantation And Death	32(28%)	22(26.1%)	6(13.6%)	

Among NDKD patients, five patients had low complement levels: two had SLE, three had post infectious glomerulonephritis. ANA positivity was seen in the two patients with SLE whereas one patient had ANCA positive vasculitis. In our study among the NDKD, acute interstitial nephritis (AIN) were 47.2% the commonest followed by membranous nephropathy - 10.4%, post infectious glomerulonephritis -10.4% and Ig A nephropathy - 7.2% and minimal change disease - 7.2%. Almost 85% of these patients - 200 were on follow up for a mean of three years and 67 of these patients required renal replacement therapy during the study period. The most common finding in patients with DKD and NDKD was AIN whereas it was post infectious glomerulonephritis in the group with isolated NDKD.

## Discussion

In our study, the frequency of NDKD in diabetic cases was 51.69%. In various studies, NDKDs also isolated or overlapped on an underlying DKD, have been described to be 8% to 85% which reflects the wide array of clinical grounds for renal biopsy [4-11]. Kittrawee et al. had taken similar inclusion criteria as our study and found NDKD to be 49% of their cases - 20% had isolated NDKD and another 29% had NDKD superimposed on DKD [12]. Considering the wide range and paucity of data from the region, it is difficult to draw any reasonable conclusion. However, this wide variation is the reason why more studies are required to establish the clinical grounds for biopsy and further evaluation in Diabetic patients presenting with renal failure.

We found that patients with a longer duration of diabetes (>5 years) and hypertension (two years) were more likely to have DKD even though they have atypical features. Similar findings were found in the study by Yenigun et al. [13], those with a duration of DM of >8+/-2 years were most likely associated with DKD. However, rather surprisingly mean blood pressure readings were not meaningfully distinguished between all the three groups (Table 2). These results may reflect the higher antihypertensive usage in the DKD group.

Similarly, unexplained renal failure which was 33.4%, and rapid worsening of renal function in diabetic patients were most likely associated with NDKD. This finding is similar to the results from Jin Kim [14], who reported that the presence of NDKD was substantially higher in patients presenting with an unexplained decline in the renal function being 20%.

Interestingly none of the other parameters including the absence of diabetic retinopathy, 24-hour proteinuria levels, presence of hypertension, and degree of hypoalbuminemia found any statistical significance in our study. A similar finding was reported by Mak et al. [15] in their study who reported no noteworthy variance in serum creatinine, albumin, glycosylated hemoglobin, and absence of retinopathy between DKD and NDKD groups. Thus it is reasonable to derive that the indicators for biopsy in diabetic patients need to be reviewed further. The research has to be targeted at finding newer criteria and probably a biomarker to differentiate DKD and NDKD.

Among the NDKD, AIN was 47.2%, the commonest cause. This finding was similar to Soni et al. [4], who reported the same as the present study followed by post-infectious glomerulonephritis and membranous nephropathy. A previous study [16] from our institution reported chronic interstitial nephritis to be the commonest cause of NDKD. However, Sharma et al. [17] reported focal segmental glomerulosclerosis (FSGS) to be the most prevalent cause of NDKD in 2,642 kidney biopsies. They reported IgA nephropathy to be the most common cause in the Asian population. The increased proportion of AIN as a cause for NDKD, compared to other studies may be explained by the increased use of OTC (over-the-counter) pain killers and antibiotics by this group of patients. An increasing incidence of AIN attributable to drug use has been documented in other studies [18]. In a retrospective analysis of community-acquired acute kidney injury (AKI), reported from an advanced care center in north India, AIN was found to be the usual routine cause of AKI in patients who underwent renal biopsy [19]. Proton pump inhibitors, commonly available in our population as an over-the-counter drug have also been speculated as a causative agent of AIN [20].

Limitations of the study being small study population and comprised regional population. Studies with larger numbers and different population groups will be helpful in authenticating the results.

## Conclusions

The clinical dilemma in differentiating DKD from NDKD still exists. The current clinical criteria do not effectively differentiate the two as the natural course of DKD in a population with type 2 DM is not well documented. From the current study, prolonged DM and hypertension of more than five years and two years, respectively, predict DKD. While unexplained renal failure was most likely with NDKD. Hence, there is a need to further refine the clinical criteria to suspect non-diabetic renal disease in diabetic patients.
